# Age-related increase in caveolin-1 expression facilitates cell-to-cell transmission of α-synuclein in neurons

**DOI:** 10.1186/s13041-021-00834-2

**Published:** 2021-07-28

**Authors:** Tae-Young Ha, Yu Ree Choi, Hye Rin Noh, Seon-Heui Cha, Jae-Bong Kim, Sang Myun Park

**Affiliations:** 1 Department of Pharmacology, Ajou University School of Medicine, 164, Worldcup-ro, Yeongtong-gu, Suwon, 16499 Korea; 2 Center for Convergence Research of Neurological Disorders, Ajou University School of Medicine, Suwon, Korea; 3 Department of Biomedical Sciences, Ajou University School of Medicine, Suwon, Korea; 4 Present Address: Department of Marine Biomedical Sciences, Hanseo University, Seosan, Chungcheongnam-do Korea

**Keywords:** Parkinson’s disease, α-synuclein, Cell-to-cell transmission, Endocytosis, Caveolin-1, Aging

## Abstract

Parkinson's disease (PD) is the second most prevalent neurodegenerative disease, with aging being considered the greatest risk factor for developing PD. Caveolin-1 (Cav-1) is known to participate in the aging process. Recent evidence indicates that prion-like propagation of misfolded α-synuclein (α-syn) released from neurons to neighboring neurons plays an important role in PD progression. In the present study, we demonstrated that cav-1 expression in the brain increased with age, and considerably increased in the brain of A53T α-syn transgenic mice. Cav-1 overexpression facilitated the uptake of α-syn into neurons and formation of additional Lewy body-like inclusion bodies, phosphorylation of cav-1 at tyrosine 14 was found to be crucial for this process. This study demonstrates the relationship between age and α-syn spread and will facilitate our understanding of the molecular mechanism of the cell-to-cell transmission of α-syn.

**Supplementary Information:**

The online version contains supplementary material available at 10.1186/s13041-021-00834-2.

## Introduction

Parkinson’s disease (PD) is the second most common age-related neurodegenerative disease after Alzheimer's disease (AD), characterized by the loss of dopaminergic neurons located in the substantia nigra pars compacta and the presence of protein inclusions called Lewy bodies or Lewy neurites, which are composed of mainly α-synuclein (α-syn), a presynaptic protein [1]. Mutations and multiplication of α-syn have been identified in early-onset familial PD [2–4], suggesting that α-syn plays a major role in the pathogenesis of PD. Furthermore, recent studies have focused on the prion-like propagation of α-syn as a novel mechanism of PD progression [5, 6].

Despite extensive research, the pathogenesis of PD remains elusive. Both genetic and environmental factors are suspected to simultaneously contribute to the pathogenesis of PD. Mutations of genes such as SNCA, parkin, PINK1, DJ-1, and LRRK2 have been identified as genetic factors of PD [7]. Among environmental factors, age is regarded as the greatest risk factor for the development of PD [8, 9].

Caveolin-1 (cav-1) is a member of the caveolin family consisting of cav-1, 2, and 3, and is known to possess diverse functions, including regulation of membrane-initiated intracellular signaling via formation of caveolae [10, 11]. Although the role of cav-1 is poorly investigated in the brain, cav-1 is widely expressed in the central and peripheral nervous systems [12], regulating neurotrophin signaling pathways and synaptic remodeling [13, 14]. Additionally, it modulates neurotransmitter receptor signaling [15, 16].

Accumulating evidence indicates that cav-1 also participates in the aging process. Reportedly, cav-1 expression is upregulated in the brain of old rats and the aged human cortex [17, 18]. Old human diploid fibroblasts express higher levels of cav-1 when compared with younger cells [19]. In human and mice, cav-1 is also upregulated in the chronologically-aged skin [20]. Additionally, cav-1-overexpressing murine embryonic fibroblasts (MEFs) display a large, flat morphology and express high levels of senescence-associated β-galactosidase activity, revealing premature cellular senescence [21]. These studies have suggested that cav-1 overexpression may induce aging phenotypes.

A previous study has shown that cells derived from parkin knock-out (KO), or downregulating parkin using shRNA, demonstrate an increase in the cav-1 level, revealing that cav-1 is a substrate for parkin [22], for which loss-of-function mutations has been identified in familial PD [23]. Furthermore, parkin KO neurons have reported an increase in the cell-to-cell transmission of α-syn, proposing that each genetic risk factor of PD can share common pathological pathways with the other factors [22]. Given that aging is the greatest risk factor for PD and cav-1 can participate in the aging process, we hypothesized that age-related cav-1 expression may affect the cell-to-cell transmission of α-syn, contributing to the pathogenesis of PD. In the present study, we explored the association between cav-1 and cell-to-cell transmission of α-syn.

## Material and methods

### Antibodies and reagents

Antibodies against cav-1 (#610407) and α-syn (#610786) were purchased from BD Biosciences (Franklin Lakes, NJ). Antibodies against human α-syn (#ab138501), Tuj-1 (#ab18207) and mCherry (#ab167453) were purchased from Abcam (Cambridge, MA). Antibodies against pCav-1 (#3251S), pSrc (Y416) (#6943), and c-Src (#2123) were purchased from Cell signaling Technology (Danvers, MA). Antibody against GAPDH (#SC-32233) was procured from Santa Cruz Biotechnology (Santa Cruz, CA). Rhodamine-conjugated transferrin (#T23365) and boron-dipyrromethene (BODIPY) FL C5-LacCer (#B34402) were purchased from Molecular Probes (Leiden, The Netherlands). Saracatinib (#11497) was procured from Cayman Chemical (Ann Arbor, MI). SKI-1 (c-Src inhibitor 1, #S2075), retinoic acid (RA) (#r2625), and bafilomycin A1 (#B1793) were obtained from Sigma (St. Louis, MO). Antibodies against Iba-1 (#019-19741) and pSer 129 α-syn (#015-25191) was purchased from Wako (Richmond, VA). Antibody against glial fibrillary acidic protein (GFAP) (#RA22101) was purchased from Neuromics (Montreal, QC). Recombinant α-syn monomers and fibrils were prepared as described previously [24]. The status of fibrils was determined by thioflavin T binding assay.

### Immunohistochemistry

C57BL/6 male mice (Orient Bio, Korea) and M83 male mice overexpressing A53T human α-syn under the control of mouse prion protein promoter (B6;C3-Tg(Prnp-SNCA*A53T)83Vle/J, The Jackson Laboratory) at indicated age were perfused, and their brains were fixed with 4% paraformaldehyde for 2 days. Frozen sections were cut at 35 μm in the coronal plane. The sections were stained using the free-floating method, adhering to the slide after staining. For 3,3’-diaminobenzidine (DAB) staining, the samples were twice rinsed with phosphate-buffered saline (PBS) containing 0.2% Triton X-100 (PBST), treated with 3% H_2_O_2_ for 5 min, and rinsed with PBST. After blocking non-specific binding by incubating with 1% bovine serum albumin (BSA) in PBST, the sections were incubated overnight at 4℃ with cav-1 antibody. Sections were rinsed three times with PBST, incubated with biotinylated secondary antibody (Vector Laboratories, Burlingame, CA) and visualized using VECTASTATIN avidin-biotin complex (ABC kit) and DAB solution (0.05% DAB and 0.003% H_2_O_2_ in 0.1 M phosphate buffer). Next, the sections were mounted on slides and images were obtained and digitalized using a Axio scan.Z1 slide scanner (Carl Zeiss, Germany) at the Three-Dimensional Immune System Imaging Core Facility of Ajou University.

### Plasmids

Plasmids for WT cav-1 and WT cav-1-EGFP were constructed using PCR. The plasmid for Y14A cav-1 was constructed using the Quick-Change site-directed mutagenesis kit (Stratagene, La Jolla, CA). Each construct was subcloned into pCDH-EF1 for generating lentiviral vectors. All plasmids were verified via DNA sequencing and prepared using the Maxi prep Kit (Qiagen, Valencia, CA).

### Cell culture

SH-SY5Y cells were grown in Dulbecco’s modified Eagle’s medium (DMEM), supplemented with 10% fetal bovine serum (FBS). Primary cortical neurons were cultured from Sprague-Dawley rat embryos or human A53T α-syn overexpressing transgenic (TG) mice at embryonic day 18, maintained in neurobasal medium (Invitrogen, Carlsbad, CA) with L-glutamine and B-27 supplement (Invitrogen, Carlsbad, CA).

### Generation of stable cell lines

WT cav-1, Y14A cav-1, WT cav-1-EGFP, and Y14A cav-1-EGFP overexpressing (OE) SH-SY5Y cells were prepared using lentiviral constructs and selected using puromycin, respectively. A53T α-syn-EGFP (A53T-E) and A53T α-syn-mCherry (A53T-M) were prepared as described previously [25]. WT cav-1/A53T-E and Y14A cav-1/A53T-E OE SH-SY5Y cells were prepared using lentiviral transfection of A53T-E in WT cav-1, Y14A cav-1 OE SH-SY5Y cells and selected using FACSAria III, respectively.

### Western blot

Cells were washed with PBS and lysed in ice-cold RIPA buffer (50 mM Tris-HCl [pH7.4], 150 mM NaCl, 0.25% sodium deoxycholate and 1% Triton X-100) containing protease inhibitor cocktail (Calbiochem, Germany) and phosphatase inhibitor cocktail (GenDEPOT, Baker, TX). After brief sonication, the lysates were incubated on ice for 20 min and centrifugated at 15,700*g* for 30 min at 4 ℃. After centrifugation, the supernatant was collected. The protein concentrations were determined with a bicinchoninic acid (BCA) protein assay kit (Bio-Rad, CA, USA). Proteins were separated by SDS-PAGE and transferred to PVDF membranes. The PVDF membrane was blocked with 1% BSA in Tris-buffered saline. After 1 h blocking, the membrane was immunoblotted with appropriate antibodies and visualized using an enhanced chemiluminescence (ECL) system (Thermo Fisher Scientific, Waltham, MA).

### Dual chamber assay

The dual chamber assay was performed as described previously [24, 25]. WT α-syn or A53T-M OE SH-SY5Y cells as donor cells were differentiated by treatment with 50 μM RA for 5 days. Next, SH-SY5Y cells or primary cortical neurons cultured on coverslips in a 12-well plate as recipient cells were cocultured with differentiated WT α-syn or A53T-M OE SH-SY5Y cells cultured on the insert for 24 h. The recipient cells were prepared for staining with anti-α-syn or anti-mCherry antibody. To measure the amount of internalized α-syn, five random fields were selected and intensities of more than 100 cells were analyzed using the MetaMorph software (Molecular Devices).

### Coculture assay

The coculture assay was performed as described previously [24, 25]. Briefly, A53T-E, WT cav-1/A53T-E or Y14A cav-1/A53T-E and A53T-M OE SH-SY5Y cells were cocultured in a 1:1 ratio for 5 days in the presence of 50 μM RA. After 5 days of coculture, the cells were then subcultured on coverslips in a 12-well plate. The cells were washed with PBS, fixed with 4% paraformaldehyde for 10 min at room temperature, washed again with PBS, stained with DAPI for 10 min, and washed again with PBS before analysis. Then, the samples were observed under a confocal microscope. To quantify double fluorescence-labeled puncta, five random fields were selected and more than 100 cells containing double fluorescence-labeled puncta were counted manually. The number of A53T-E OE SH-SY5Y cells containing double fluorescence labeled puncta and the number of A53T-M OE SH-SY5Y cells containing double fluorescence-labeled puncta were counted separately and expressed as percentages of total cells analyzed.

### Microfluidic chamber assay

Microfluidic chamber assay was performed as described previously with slight modification [26]. Briefly, triple compartment microfluidic devices (TCND1000) were obtained from Xona Microfluidic, LLC (Temecula, CA, USA). Glass coverslips were prepared and affixed to the microfluidic device according to the manufacturers’ instructions. Approximately 100,000 A53T TG cortical neurons (TP18) were plated per chamber. At 7 DIV, 0.5 μg of α-syn fibrils were added into chamber (C) 1, with lentiviruses for EGFP-only, WT cav-1-EGFP or Y14A cav-1-EGFP expression added into C2. To control the direction of flow, a 50 μl difference in media volume was maintained between C1 and C2, and C2 and C3, according to the manufacturers’ instructions. Neurons were fixed at 7 days after treatment with α-syn fibrils using 4% paraformaldehyde in PBS. Devices were then removed, and immunofluorescence assay was performed.

### Confocal microscopy

Cells cultured on poly-D-lysine coated coverslips were washed twice with PBS and fixed in 4% paraformaldehyde for 10 min at room temperature. The fixed cells were then washed with PBS and incubated with PBS containing 0.1% Triton X-100 for 10 min at room temperature. After washing several times with PBS, cells were blocked with PBS containing 1% BSA for 1 h at room temperature, and then incubated overnight with indicated antibodies at 4 °C. The samples were then stained with fluorescence conjugated secondary antibodies for 1 h, mounted with VECTASHIELD (Vector Laboratories, Burlingame, CA), and observed under a confocal microscope (Zeiss, Germany).

### Statistical analysis

All values are expressed as the mean ± SEM. Statistical significance was evaluated using Graphpad Prism (La Jolla, CA).

## Results

### The level of cav-1 expression increases in the brain with age

**Fig. 1:**
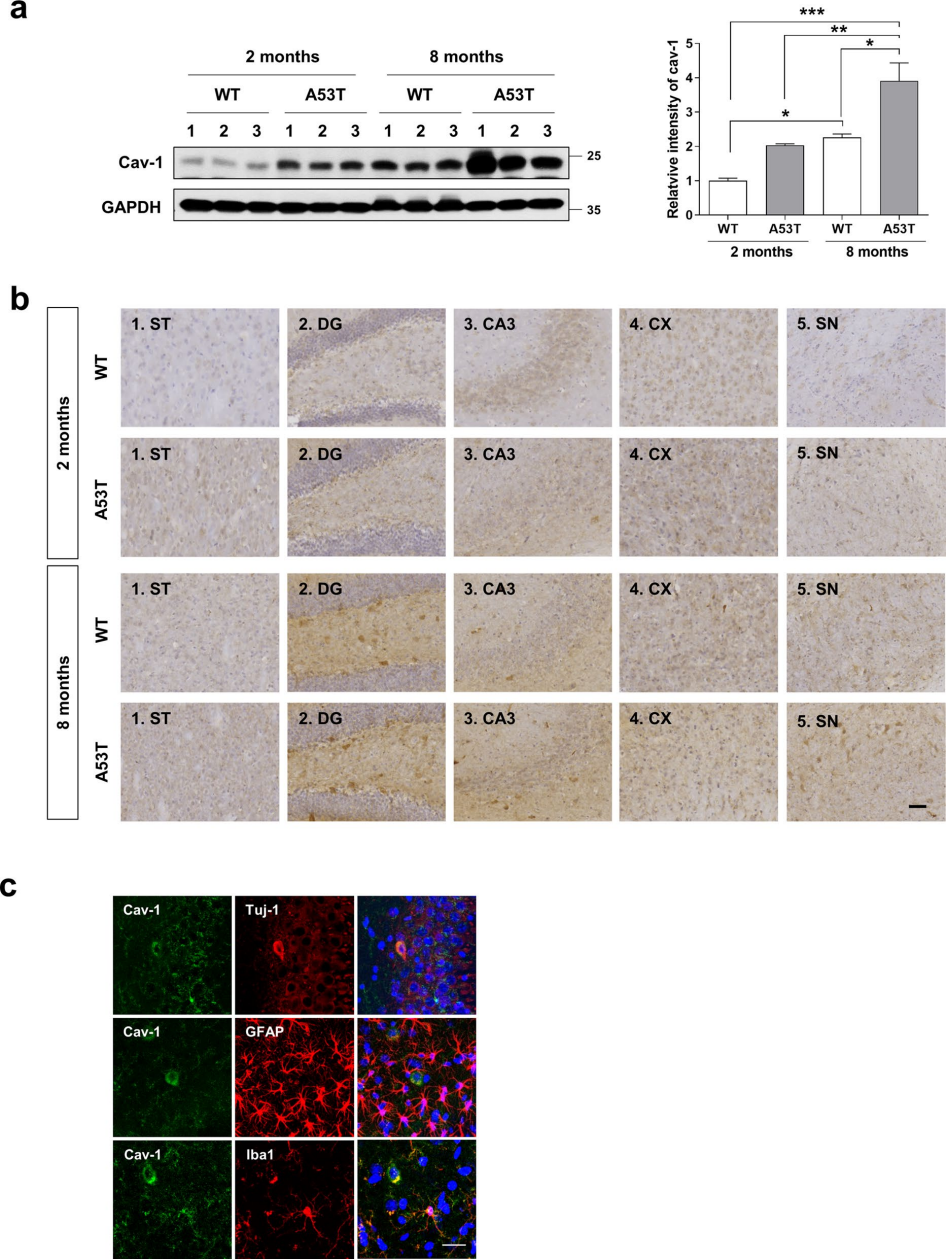
Cav-1 expression increases in the brain with age. **a** Brain hemispheres obtained from WT and A53T transgenic (TG) mice at the age of 2 and 8 months were lysed and Western blot was performed with indicated antibodies. Values were derived from three individual mice (n = 3). *p < 0.05, **p < 0.01, ***p < 0.001, one-way ANOVA. **b** Immunohistochemistry for cav-1 at 2 months or 8 months was performed in WT or A53T TG mice. Images were derived from more than three individual mice. Scale bar indicates 50 μm. ST, triatum; DG, dentate gyrus; CX, cortex; SN, substantia nigra. **c** Brain tissues containing the hippocampus from A53T TG mice at the age of 8 months were immunostained with indicated antibodies and observed under a confocal microscope. Scale bar indicates 20 μm.

As cav-1 expression is reportedly related to aging [17, 19, 20], we first explored the age-related increase in cav-1 protein expression in mice. We observed that cav-1 protein expression was increased in whole brain lysates of mice in an age-dependent manner (Fig. 1a), consistent with a previous study [17]. Immunohistochemical analysis showed that cav-1 protein was widely expressed in the brain. Especially, cav-1 protein was predominantly expressed in the hippocampus when compared with the cortex, striatum, substantia nigra. Furthermore, the expression of cav-1 protein was increased with age (Fig. 1b). Additionally, we compared cav-1 protein expression between wild-type (WT) and A53T α-syn transgenic (TG) mice, a PD model demonstrating an improved simulation of PD progression as observed in patients [27]. Compared with WT mice at both ages, cav-1 protein was higher in A53T α-syn TG mice (Fig. 1a–b). In addition, cav-1 protein expression was increased mainly in neurons and microglia, but not astrocytes (Fig. 1c). These results suggest that cav-1 protein expression was increased in the brain with age, and A53T α-syn overexpression facilitated cav-1 protein expression in the brain.

### The uptake of ɑ-syn is increased in cav-1 overexpressing SH-SY5Y cells and rat primary cortical neurons

**Fig. 2:**
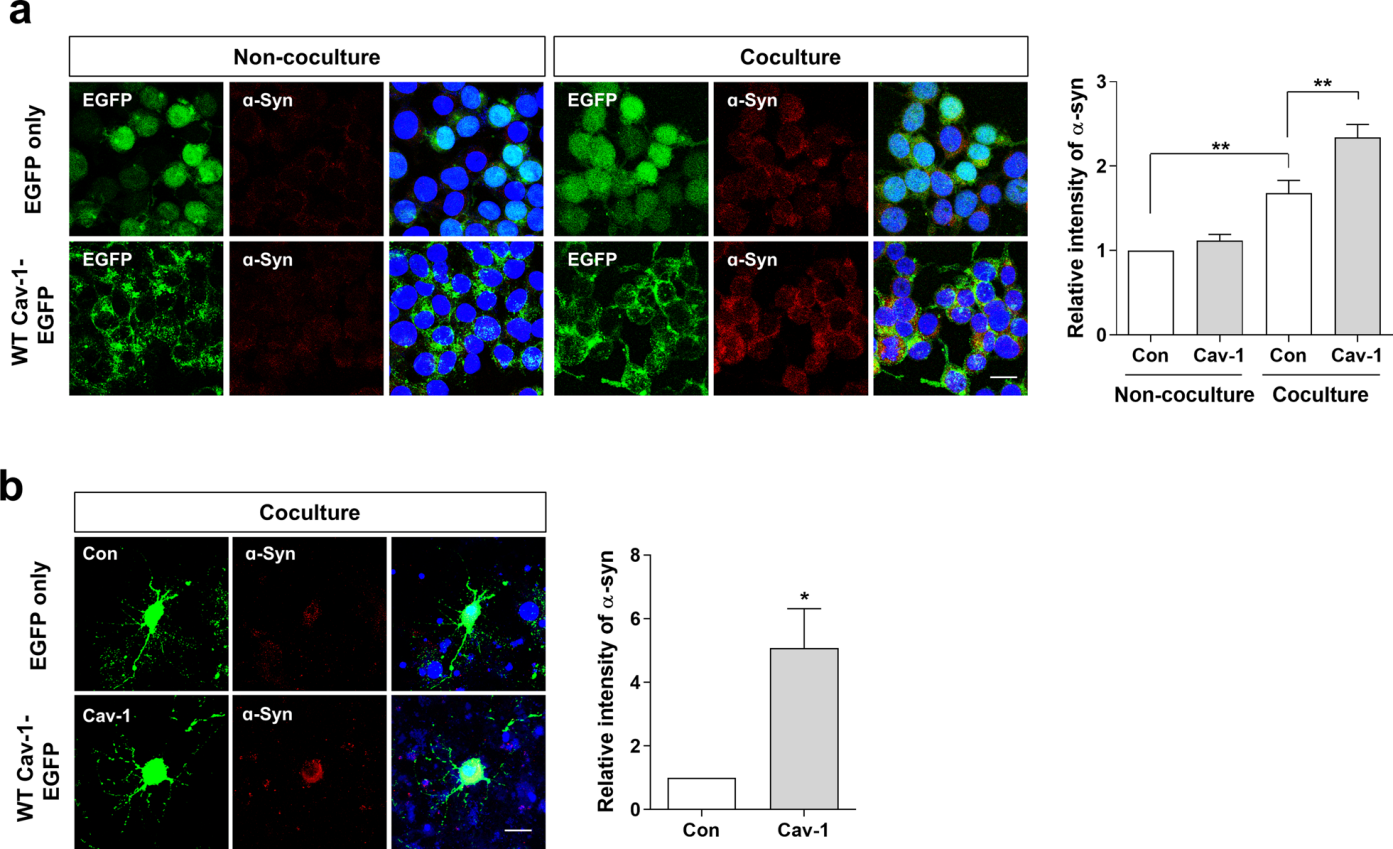
The uptake of α-syn increases in cav-1 overexpressing SH-SY5Y cells and rat primary cortical neurons. **a** EGFP only (Con) and WT cav-1-EGFP OE SH-SY5Y cells (Cav-1) were cocultured with differentiated α-syn OE SH-SY5Y cells cultured on the insert for 24 h. The samples were fixed and immunostained with α-syn antibody (BD) and observed under a confocal microscope. The intensity of five independent experiments was analyzed. **b** Rat primary cortical neurons were transfected with EGFP and WT cav-1-EGFP plasmids. After 1 day of transfection, the cells were cocultured with differentiated α-syn OE SH-SY5Y cells cultured on the insert for 24 h. The samples were fixed and immunostained with human α-syn antibody (Abcam) and observed under a confocal microscope. The intensity of four independent experiments was analyzed. Blue indicates DAPI staining. Scale bars indicate 20 μm. *p < 0.05, **p < 0.01, one-way ANOVA (**a**), unpaired t-test (**b**)

Next, we investigated the effect of cav-1 expression on the uptake of α-syn using a dual chamber system, which efficiently monitors cell-derived α-syn uptake into cells [24, 25]. We generated EGFP only or WT cav-1-EGFP overexpressing (OE) SH-SY5Y stable cell lines, EGFP only and WT cav-1-EGFP were confirmed to be well-expressed (Additional file 1: Fig. S1a–b). As shown in Fig. 2a, α-syn was rarely detected in SH-SY5Y cells overexpressing EGFP only or WT cav-1-EGFP without coculture, whereas α-syn was detected in both cells by coculturing with α-syn OE SH-SY5Y cells. Moreover, α-syn levels were higher in SH-SY5Y cells overexpressing WT cav-1-EGFP than in SH-SY5Y cells overexpressing EGFP only. Western blot further confirmed it (Additional file 1: Fig. S1c). Rat primary cortical neurons overexpressing cav-1 revealed similar results (Fig. 2b), suggesting that the uptake of cell-released α-syn was increased following cav-1 overexpression.

### Phosphorylation of cav-1 at tyrosine 14 is important to regulate the uptake of ɑ-syn

**Fig. 3:**
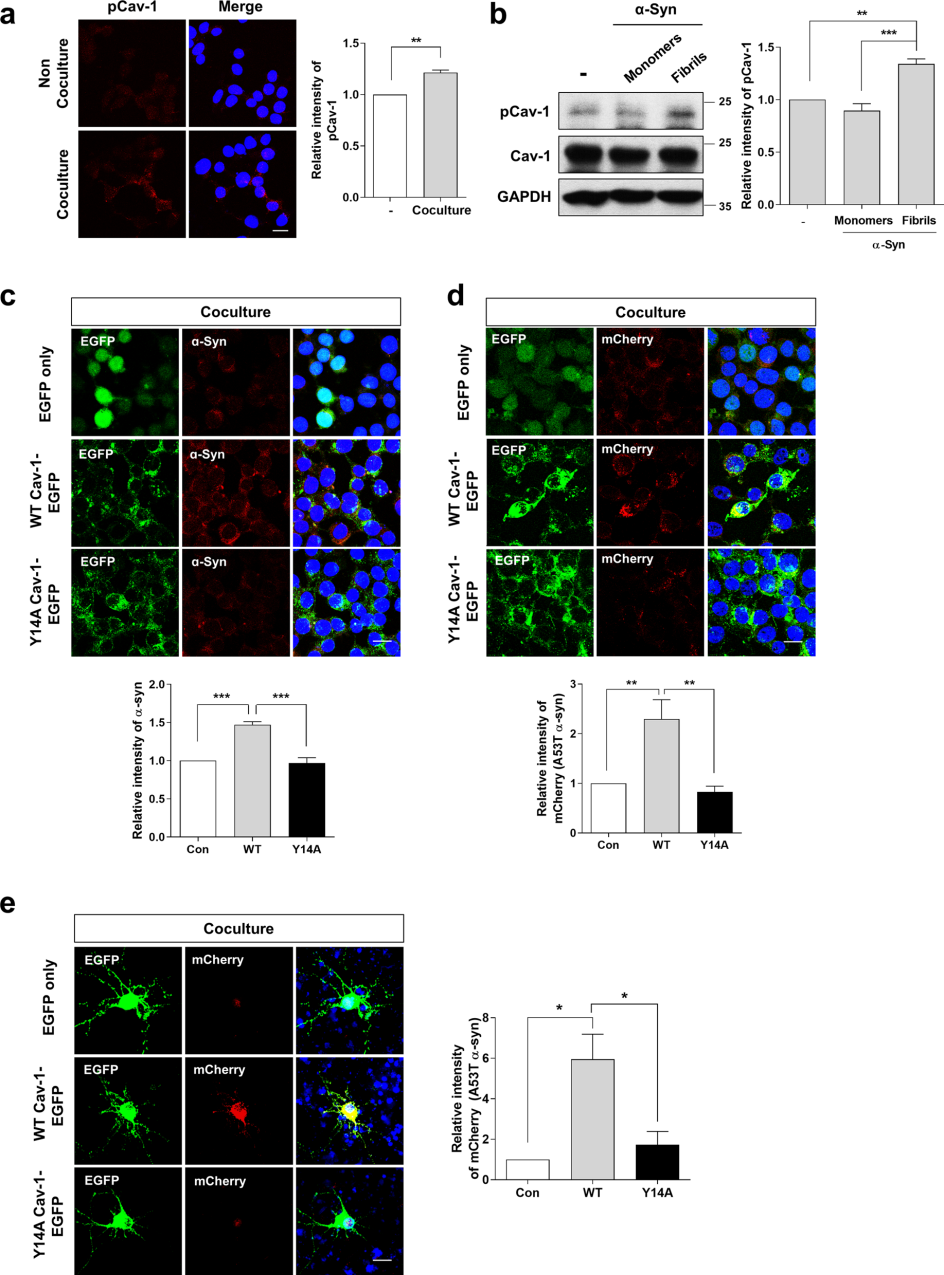
Phosphorylation of cav-1 at tyrosine 14 is important to regulate the uptake of α-syn. **a** WT cav-1 OE SH-SY5Y cells were cocultured with differentiated α-syn OE SH-SY5Y cells cultured on the insert for 24 h. The samples were fixed and immunostained with pCav-1 antibody and observed under a confocal microscope. The intensity of three independent experiments was analyzed. **b** WT cav-1 OE SH-SY5Y cells were incubated in the presence of 1 μM α-syn monomers or α-syn fibrils (n = 5) for 10 min. The cells were then lysed, and Western blot was performed with the indicated antibodies. The band intensity of four independent experiments was analyzed. **c** Each stable cell line expressing EGFP only (Con), WT cav-1-EGFP (WT) and Y14A cav-1-EGFP (Y14A) was cocultured with differentiated α-syn OE SH-SY5Y cells for 5 days using a dual-chamber system (n = 5). After 24 h of coculture, the samples were stained with α-syn antibody (BD). **d** Each stable cell line was cocultured with differentiated A53T α-syn-mCherry OE SH-SY5Y cells (A53T-M) using the dual-chamber system (n = 4). After 24 h of coculture, the samples were stained with mCherry antibody. **e** Rat primary cortical neurons were transfected with EGFP, WT cav-1-EGFP and Y14A cav-1-EGFP plasmids. After one day of transfection, neurons were cocultured with A53T-M OE SH-SY5Y cells for 24 h (n = 3). The cells were stained with mCherry antibody. All images are observed under a confocal microscope. Blue indicates DAPI staining. Scale bars indicate 20 μm. The intensity of independent experiments was analyzed. *p < 0.05, **p < 0.01, ***p < 0.001, unpaired t-test (**a**), one-way ANOVA (**b**–**e**)

Cav-1 is a phosphoprotein, and the tyrosine 14 residue of cav-1 is considered the principal site for tyrosine phosphorylation [28–30]. We investigated whether the phosphorylation of cav-1 is involved in the uptake of α-syn. pCav-1 was increased in WT cav-1 OE SH-SY5Y cells by coculturing with α-syn OE SH-SY5Y cells (Fig. 3a). Variable forms of α-syn are released from cells [31–33]. Y14 of cav-1 was phosphorylated following treatment with α-syn fibrils, but not with monomeric α-syn (Fig. 3b), suggesting that α-syn fibrils induced the phosphorylation of cav-1. Next, we generated a cav-1 mutant stable cell line overexpressing Y14A cav-1-EGFP, which was confirmed by Western blot against the phosphorylation of cav-1 (Additional file 1: Fig. S2a). There was also no difference in intracellular localization between WT and Y14A cav-1 (Additional file 1: Fig. S1b). α-Syn uptake was increased in SH-SY5Y cells overexpressing WT cav-1-EGFP, but not in cells overexpressing Y14A cav-1-EGFP (Fig. 3c). On coculturing these cells with A53T α-syn-mCherry (A53T-M) OE SH-SY5Y cells using a dual chamber and staining with anti-mCherry antibody to rule out the possibility of detecting small amount of α-syn expressed endogenously, we observed similar results (Fig. 3d). Moreover, while the uptake of A53T-M was increased in primary neurons overexpressing WT cav-1-EGFP, this was not observed in cells overexpressing Y14A cav-1-EGFP (Fig. 3e). These results suggested that the phosphorylation of cav-1 at the tyrosine 14 residue is important for α-syn uptake.

**Fig. 4:**
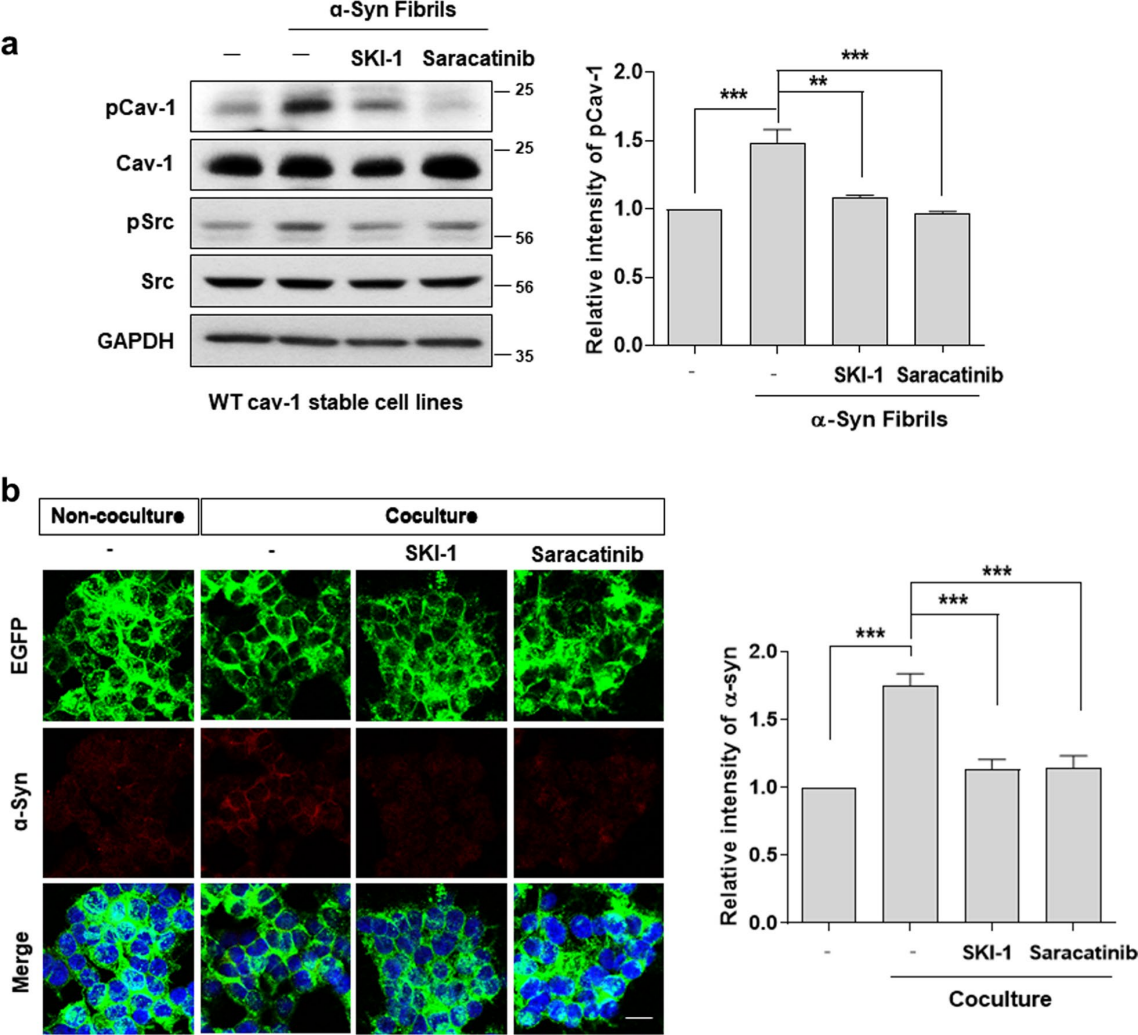
The phosphorylation of cav-1 is increased by α-syn fibrils and decreased by c-Src inhibitors. **a** WT cav-1 stable cell line was treated with 1μM α-syn fibrils for 10 min. Before treatment with α-syn fibrils, 10 μM SKI-1 or 0.1 μM saracatinib were pretreated for 30 min. Each lysate was then analyzed using Western blot. The band intensity of three independent experiments was quantified. **b** The WT cav-1-EGFP cell line was cocultured with α-syn OE SH-SY5Y cell using the dual-chamber system for 24 h in the presence of 10 μM SKI-1 or 0.1 μM saracatinib. The cells were stained with α-syn antibody (BD) and observed using confocal microscopy. Blue indicates DAPI staining. Scale bar indicates 20 μm. The intensity values are obtained from five independent experiments. **p < 0.01, ***p < 0.001, one-way ANOVA (**a**–**b**)

Reportedly, Y14 of cav-1 is mainly phosphorylated by Src tyrosine kinases [28–30]. To confirm the importance of cav-1 phosphorylation in α-syn uptake, we utilized the Src kinase inhibitor, SKI-1 (c-Src inhibitor 1), and saracatinib (c-Src/Abl dual kinase inhibitor). The increased Y14 phosphorylation of cav-1 by α-syn fibrils was significantly reduced by SKI-1 and saracatinib (Fig. 4a). Furthermore, the uptake of cell-derived α-syn into SH-SY5Y cells overexpressing WT cav-1 was attenuated following treatment with SKI-1 or saracatinib (Fig. 4b), suggesting that cav-1 was phosphorylated by Src kinases and the inhibition of c-Src activity attenuated α-syn uptake.

### The phosphorylation of cav-1 at tyrosine 14 residue regulates lipid rafts dependent endocytosis.

**Fig. 5:**
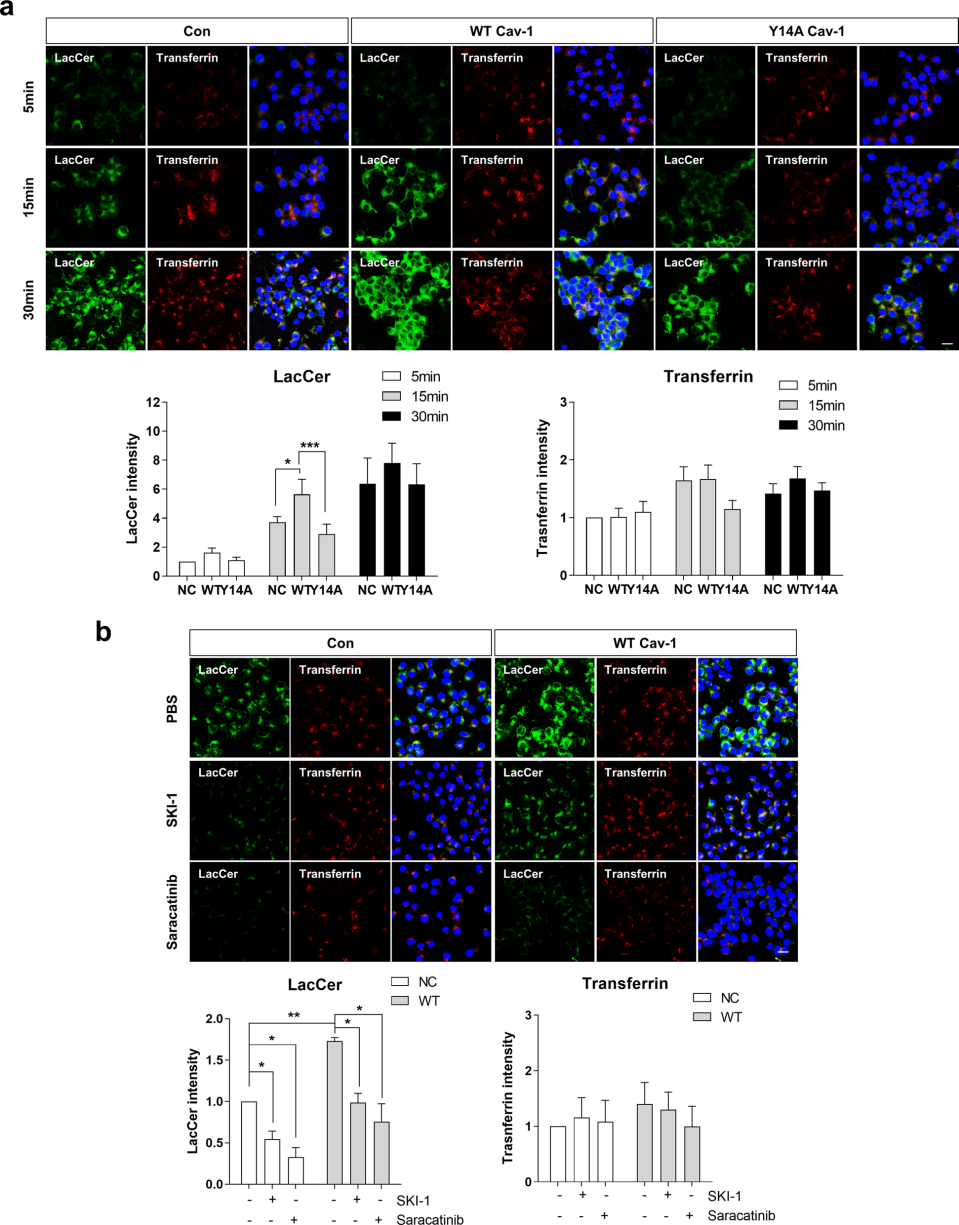
The phosphorylation of cav-1 regulates lipid rafts dependent endocytosis. **a** Each cell line was incubated with 50 nM BODIPY FL C5-Lactosylceramide (LacCer) (Green) and 2.5 μg/ml rhodamine-conjugated transferrin (Red) for 5, 15 and 30 min. **b** Each cell line was pretreated with 10 μM SKI-1 or 0.1 μM saracatinib for 30 min and further incubated with 50 nM BODIPY FL C5-Lactosylceramide (LacCer) (Green) and 2.5 μg/ml rhodamine-conjugated transferrin (Red) for 20 min. Next, the cells were fixed and observed via confocal microscopy. The intensity of five (**a**) or four (**b**) independent experiments were quantified. Blue indicates DAPI staining. Scale bars indicate 20 μm. *p < 0.05, ***p < 0.001, two-way ANOVA

It has been reported that cav-1 stimulates lipid rafts-dependent endocytosis [34]. Accordingly, to investigate whether the Y14 phosphorylation of cav-1 regulates endocytosis, we performed an in vitro endocytosis assay using LacCer as a marker of lipid rafts-dependent endocytosis [35] and transferrin as a marker of clathrin-dependent endocytosis [36]. As shown in Fig. 5a, LacCer uptake was transiently accelerated in WT cav-1 OE SH-SY5Y cells, compared with control cells, but not in Y14A cav-1 OE SH-SY5Y cells. In contrast, the level of transferrin uptake did not differ among cells. LacCer uptake was also decreased in the presence of c-src inhibitors (Fig. 5b), suggesting that cav-1 regulates lipid rafts-dependent endocytosis via cav-1 phosphorylation at tyrosine 14.

### Cav-1 induces the uptake of ɑ-syn regardless of the degradation pathway

**Fig. 6:**
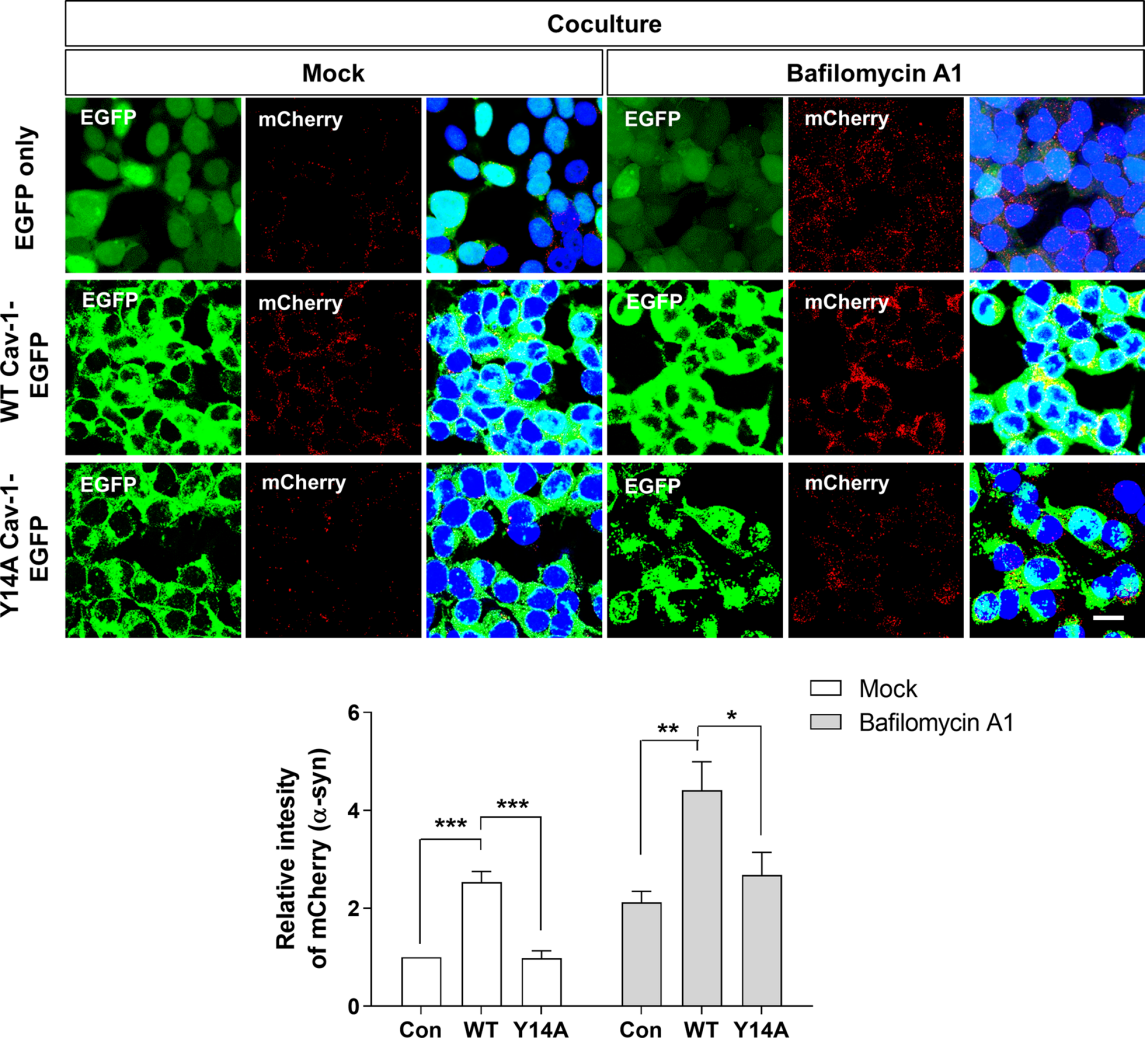
Cav-1 induces the uptake of α-syn regardless of the degradation pathway. Each cell line was cocultured with differentiated A53T-M OE SH-SY5Y cells in the absence or presence of 50 nM bafilomycin A1 using the dual-chamber system. After 24 h of coculture, the samples were stained with mCherry antibody. Scale bar indicates 20 μm. Blue indicates DAPI staining. The intensity was analyzed from five independent experiments. * p < 0.05, **p < 0.01, ***p < 0.001, one-way ANOVA

To confirm whether the increased levels of transferred α-syn in cav-1 OE cells could be attributed to the enhanced uptake of transferred α-syn into cells, or limited degradation of transferred α-syn in recipient cells via defects in the protein degradation system such as autophagy, a dual chamber assay was performed in the presence of bafilomycin A1, a well-known autophagy inhibitor. Despite the presence of bafilomycin A1, higher amounts of transferred α-syn were detected in WT cav-1-EGFP OE SH-SY5Y cells, when compared with control or Y14A cav-1-EGFP OE SH-SY5Y cells, although the relative amounts of detected transferred α-syn were increased (Fig. 6). This suggested that the higher amounts of transferred α-syn, detected in WT cav-1 OE SH-SY5Y cells, could be attributed to enhanced α-syn uptake, but not an impaired degradation system.

### Formation of Lewy body-like inclusions is increased by WT cav-1 overexpression and attenuated by Y14A cav-1 overexpression

**Fig. 7:**
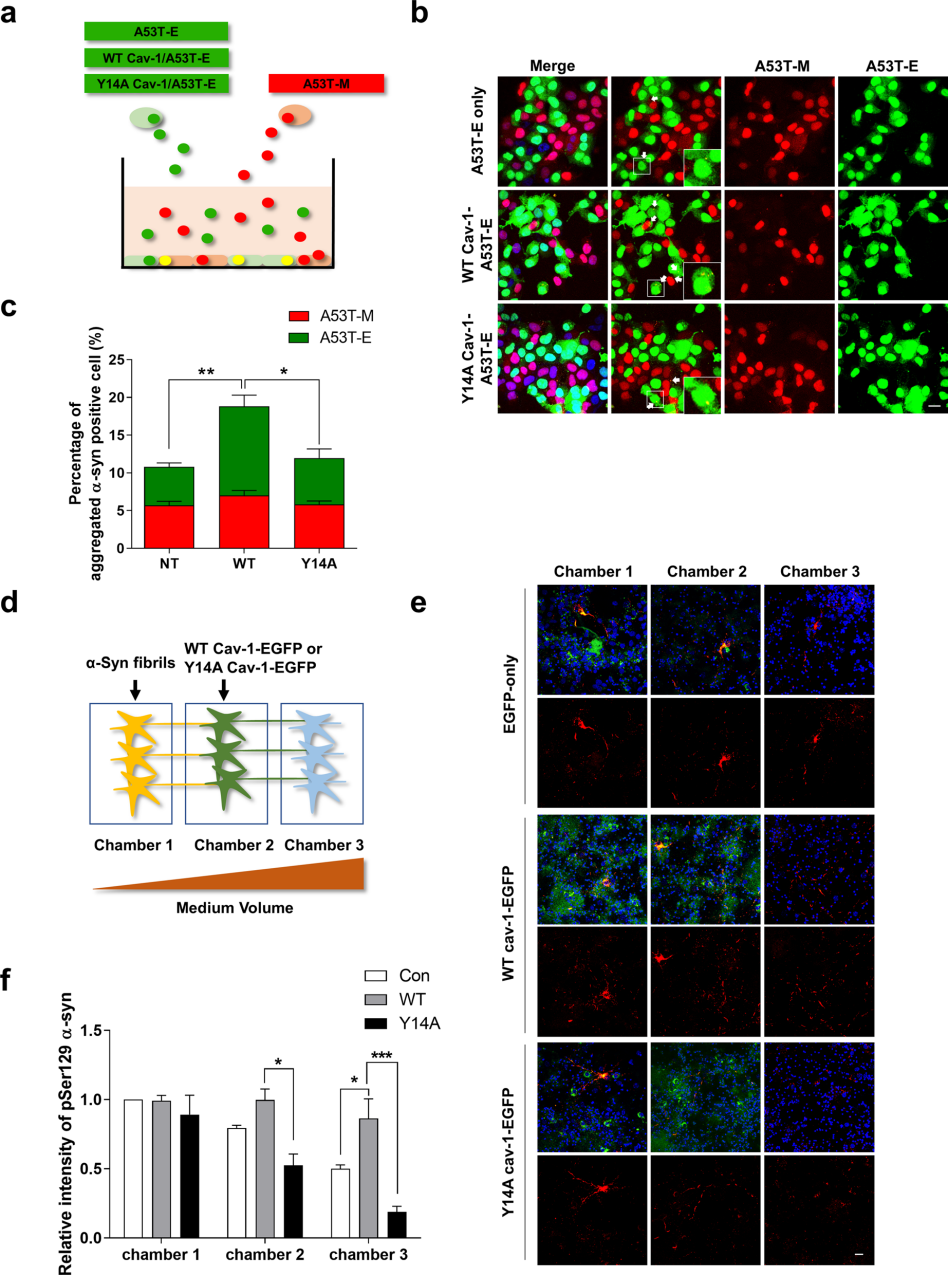
Cav-1 mediates Lewy body-like inclusion body formation. **a** Schematic diagram showing coculture assay. **b**–**c** A53T-E, WT cav-1-A53T-E and Y14A cav-1-A53T-E OE SH-SY5Y cells were cocultured with A53T-M OE SH-SY5Y cells for 5 days in the presence of 50 μM RA. Next, the samples were observed under a confocal microscope, and the number of cells containing double fluorescence-labeled puncta was analyzed. Arrows indicate double fluorescence-labeled puncta. In the graphs, green indicates the number of A53T-E cells, containing double fluorescence-labeled puncta, and red indicates the number of A53T-M cells, containing double fluorescence-labeled puncta. The values obtained are from five independent experiments. **d** Schematic diagram showing the microfluidic chamber assay. **e** Primary neurons from A53T TG mice were cultured in microfluidic chambers. At 7 DIV, 0.5 μg of α-syn fibrils were added into chamber 1, and lentiviruses of EGFP-only, WT cav-1-EGFP or Y14A cav-1-EGFP were added into chamber 2. Neurons were fixed at 7 days after treatment with α-syn fibrils. The devices were then removed. The cells were immunostained with pSer129 α-syn antibody. **f** The intensity was analyzed. Values were derived from four independent experiments. *P < 0.05, **p < 0.01, ***P < 0.001, one-way ANOVA. Blue indicates DAPI staining. Scale bars indicate 20 μm

In a previous study, we developed an in vitro model system to explore the inclusion body formation by the cell-to-cell transmission of α-syn. In this model system, double fluorescence-labeled aggregation puncta are colocalized with pSer129 α-syn, which is abundantly found in Lewy body [37], suggesting that double fluorescence-labeled aggregation puncta are Lewy body-like inclusions [25]. To determine whether cav-1 expression regulates the inclusion body formation through the cell-to-cell transmission of α-syn, we generated stable cell lines overexpressing WT cav-1 or Y14A cav-1 with A53T α-syn-EGFP (A53T-E). These cells expressed A53T α-syn-EGFP similarly (Additional file 1: Fig. S2b) and were cocultured with A53T-M OE SH-SY5Y cells for 5 days in the presence of RA to induce differentiation (Fig. 7a). After 5 days of coculture, we counted cells containing double fluorescence-labeled aggregation puncta (yellow puncta) that were generated by the aggregation of both α-syn transferred from neighboring cells and expressed α-syn inside cells. The number of cells containing yellow puncta was increased in A53T-E OE SH-SY5Y cells overexpressing WT cav-1, but not in A53T-M OE SH-SY5Y cells. In contrast, the number of cells containing yellow puncta did not increase in A53T-E OE SH-SY5Y cells overexpressing Y14A cav-1 (Fig. 7b–c). Additionally, for further validation, we used microfluidic culture devices developed to monitor the cell-to-cell transmission of α-syn in primary neurons (Fig. 7d) [26]. Following addition of α-syn fibrils to Chamber 1 (C1) neurons with further incubation for 7 days, α-syn pathology were detected in C2 and C3 neurons. Next, we overexpressed WT cav-1 in C2 neurons. The addition of α-syn fibrils to C1 induced a higher α-syn pathology in C2 and C3 neurons, but not through Y14A cav-1 overexpression (Fig. 7e–f), suggesting that cav-1 is involved in the formation of Lewy body-like inclusions via the uptake of α-syn and those are regulated by Y14A phosphorylation of cav-1.

## Discussion

PD is a disabling neurodegenerative disorder that is strongly associated with aging [38, 39]. At the cellular level, postmortem tissues from sporadic patients with PD displayed an increased expression of senescent markers, including p16INK4a, and several senescence-associated secretory phenotype (SASP) factors, matrix metalloproteinase-3 (MMP-3), interleukin (IL)-6, IL-1a, and IL-8 [40]. Additionally, elevated p16INK4a and MMP-3 expression have been reported in cortical tissues of patients with AD [41], suggesting that the basic mechanisms of aging may be closely related to the pathogenesis of neurogenerative diseases such as AD and PD.

Reportedly, cav-1 expression has been associated with aging. Several previous studies using in vitro senescent cells [19] and in vivo aged tissues [17, 18] have suggested that cav-1 expression is increased with aging. Additionally, cav-1 overexpression displays senescence phenotypes [21, 42, 43], suggesting that increased cav-1 expression may play an important role in aging at the cellular level, although there have been conflicting observations that cav-1 knockout accelerates premature senescence in MEFs [44] and loss of cav-1 accelerates neurodegeneration and aging [45].

In the present study, we observed that cav-1 expression was increased in the brain in an aging-dependent manner, which is consistent with previous studies [17, 18]. Moreover, cav-1 expression was considerably elevated in the brain of A53T TG mice. In patients with PD, homozygous haplotypes have been observed upstream of human cav-1, which induce increased gene expression [46]. In vitro, overexpression of α-syn upregulates cav-1 expression [47, 48], supporting our data that demonstrates the association of elevated cav-1 expression with PD.

Accumulating evidence suggests that the mechanism of pathogenic α-syn spreading throughout the nervous system underlies the pathogenesis of PD [49], which remains poorly understood. We observed that cav-1 overexpression in neurons accelerated α-syn uptake into neurons and inclusion body formation via the cell-to-cell transmission of α-syn. It is well known that cav-1 regulates caveolae-dependent endocytosis [50]. Classical caveolae-mediated endocytosis may not occur in neurons as neurons lack caveolae [51, 52]. Nevertheless, a previous study has suggested that cav-1 also demonstrates caveolae-independent functions including trafficking of proteins to and from the plasma membrane [53]. Furthermore, α-syn is internalized into cells via various mechanisms in a species-dependent manner [54–56]. Accordingly, neuronal cav-1 may be involved in α-syn uptake via caveolin-dependent endocytosis. Our hypothesis was supported by the observation that cav-1 overexpression accelerates lipid rafts-dependent endocytosis, but not clathrin-dependent endocytosis. Interestingly, exogenously added α-syn fibrils induce lipid rafts-dependent endocytosis [25], with numerous PD-associated gene products, including parkin, DJ-1, and UCH-L1 also regulating lipid rafts-dependent endocytosis [22, 57, 58], indicating that the dysfunction of lipid rafts-dependent endocytosis may be associated with the pathogenesis of PD as a common pathological mechanism.

The activity of the degradation pathways, both autophagy and proteasome-mediated, are reduced during aging [59, 60]. Reportedly, cav-1 overexpression prevents autophagy in human osteosarcoma cells [61] and cav-1 deletion increases basal autophagy in the vascular endothelium [62]. Considering that α-syn is degraded by autophagy [63–65], we further investigated whether our finding that increased α-syn uptake into cav-1 OE cells resulted from enhanced cellular uptake of transferred α-syn or limited degradation of transferred α-syn following bafilomycin A1 treatment. Relative amounts of transferred α-syn detected in recipient cells were increased in the presence of bafilomycin A1. Inhibition of α-syn degradation induces the accumulation of α-syn, further releasing α-syn into the extracellular space via different secretory pathways [66–68]. Accordingly, α-syn accumulation could be attributed to increased α-syn release by inhibiting the autophagic degradation of α-syn in donor cells, and/or increased accumulation of α-syn in recipient cells. Nevertheless, α-syn was highly detected in cav-1 OE cells, suggesting that cav-1 overexpression enhanced the uptake of transferred α-syn into cells, although we were unable to comprehensively elucidate whether cav-1 overexpression affected the autophagy system to degrade transferred α-syn in our experimental condition.

**Fig. 8:**
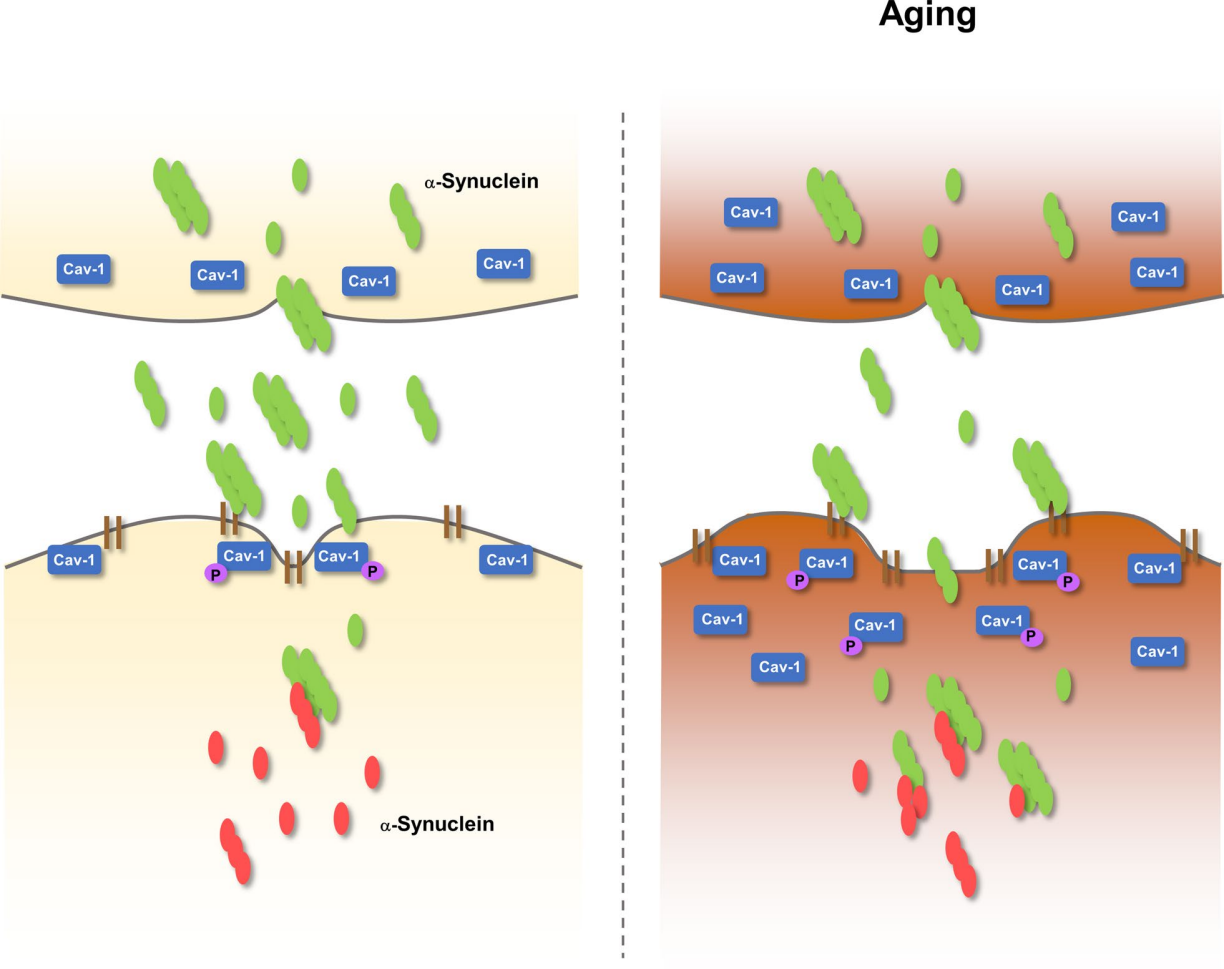
Proposed model of the association of cav-1 in the uptake of α-syn. Cav-1 expression increases in the brain with age. Increased cav-1 expression facilitates the uptake of extracellular α-syn, as well as additional Lewy body-like inclusion formation with expressed α-syn inside cells. The phosphorylation of cav-1 is involved in the uptake of α-syn

Given that cav-1 expression increases with age [17, 18] and cav-1 overexpression induces cellular senescence [21, 42, 43], and our observation that cav-1 overexpression accelerated α-syn uptake into neurons and inclusion body formation, α-syn propagation may be further accelerated in aging individuals (Fig. 8), which is consistent with a previous study revealing that aging-promoting genetic variations accelerate the rate of cell-to-cell transmission of α-syn aggregates in a C. elegans model [69].

The phosphorylation of cav-1 at the tyrosine 14 residue is related to macromolecular transcytosis as a specific form of the scaffold to recruit and organize multiple molecular components of the transcytotic machinery [70, 71]. Src-mediated phosphorylation of caveolin-1 Tyr-14 is necessary for caveolar endocytosis of EGFR under oxidative stress [72]. Accordingly, tyrosine 14 phosphorylation of cav-1 may regulate endocytosis in neurons. Furthermore, we observed that Y14A cav-1 overexpression failed to accelerate α-syn uptake into neurons, as well as inclusion body formation. Y14A cav-1 overexpression also failed to enhance LacCer uptake as a representative molecule for lipid rafts-dependent endocytosis, suggesting that the phosphorylation of cav-1 at the tyrosine 14 residue in neurons plays an important role in cell-to-cell transmission of α-syn by regulating lipid rafts-dependent endocytosis.

As cav-1 was first identified as a major phosphorylated protein in v-Src-expressing cells [73], it has been well documented that the tyrosine 14 residue of cav-1 is phosphorylated by Src kinases [29, 30, 74]. We observed that the phosphorylation of cav-1 at tyrosine 14 was induced by α-syn fibrils, but not by α-syn monomers. Moreover, the phosphorylation of cav-1 at tyrosine 14 induced by α-syn fibrils was dependent on c-Src kinase activity. Additionally, the inhibition of cav-1 phosphorylation by regulating c-Src activity attenuated the accelerated α-syn uptake by cav-1 overexpression. Previously, we have demonstrated that α-syn fibrils bind to FcγRIIB expressed in neurons and stimulate the cell-to-cell transmission of α-syn via SHP-1/-2 as a downstream mediator of FcγRIIB signaling [25]. Furthermore, the activation of SHP-1/-2 by α-syn fibrils stimulates c-Src phosphorylation, accelerating the cell-to-cell transmission of α-syn [24]. Accordingly, cav-1 may function as a downstream mediator of FcγRIIB-SHP-1/-2-c-Src for α-syn uptake.

Inhibiting cav-1 upregulation with selective cyclooxygenase (COX)-2 inhibitors attenuates the development of cellular senescence in human dermal fibroblasts [75]. Furthermore, an inhibitor of phosphatidylcholine-specific phospholipase C reduces the upregulation of cav-1 expression, as well as the number of replicative senescent bone marrow stromal cells [76]. Daidzein, known to inhibit cav-1 expression, restores memory deficits in an intracerebroventricular streptozotocin (ICV-STZ)-induced neurodegeneration rat model [77]. Additionally, our data support the postulation that inhibiting the upregulation of cav-1 expression or cav-1 phosphorylation can attenuate the progression of PD, as well as the aging process. In agreement with our hypothesis, reduced cav-1 expression reportedly extended lifespan and mitigated toxic protein aggregation by modulating the expression of age regulating and signaling-promoting genes [78].

## Conclusions

We demonstrated that cav-1 expression was increased in the brain with age. Cav-1 overexpression in neurons increased cell-to-cell transmission of α-syn. Inhibiting cav-1 phosphorylation attenuated the cell-to-cell transmission of α-syn, further suppressing Lewy body-like inclusion body formation. This study will help elucidate the molecular mechanism of intercellular α-syn transmission, as well as to develop new therapeutics against PD.

## Supplementary Information

**Additional file 1: Figure S1. **Protein expression of stable cell line and the uptake of α-syn in cav-1 overexpressing SH-SY5Y cells. **a **EGFP only, WT cav-1-EGFP and Y14A cav-1-EGFP OE SH-SY5Y cells were lysed, and Western blot was performed with the indicated antibodies. **b **EGFP only, WT cav-1-EGFP and Y14A cav-1-EGFP OE SH-SY5Y cells were observed under confocal microscopy. **c **EGFP only and WT cav-1-EGFP OE SH-SY5Y cells were cocultured with differentiated α-syn OE SH-SY5Y cells cultured on the insert for 24 h. The cells were then lysed, and Western blot was performed with the indicated antibodies. The intensity of four independent experiments was analyzed. ** p < 0.01, unpaired t-test. **Figure S2. **Protein expression of stable cell line.** a **EGFP only, WT cav-1-EGFP or Y14A cav-1-EGFP OE SH-SY5Y cells were treated with 1 μM α-Syn fibrils for 10 min. The cells were then lysed, and Western blot was performed with the indicated antibodies. **b **A53T-E only, WT cav-1/A53T-E and Y14A cav-1/A53T-E OE SH-SY5Y cells were lysed, and Western blot was performed with the indicated antibodies.

## Data Availability

The datasets used and/or analyzed during the current study available from the corresponding author on reasonable request.

## Abbreviations

PD: Parkinson's disease
AD: Alzheimer's disease
cav-1: Caveolin-1
α-syn: α-synuclein
MEFs: Murine embryonic fibroblasts
KO: Knock-out
DAB: 3,3’-diaminobenzidine
PBS: Phosphate-buffered saline
PBST: PBS containing 0.2% Triton X-100
BSA: Bovine serum albumin
ABC: Avidin-biotin complex
DMEM: Dulbecco’s modified Eagle’s medium
FBS: Fetal bovine serum
A53T-E: A53T α-syn-EGFP
A53T-M: A53T α-syn-mCherry
OE: Overexpressing
BCA: Bicinchoninic acid
ECL: Chemiluminescence
C: Chamber
TG: Transgenic
SASP: Senescence-associated secretory phenotype
MMP-3: Matrix metalloproteinase-3
IL: Interleukin
COX: Cyclooxygenase
ICV-STZ: Intracerebroventricular streptozotocin
LacCer: BODIPY FL C5-Lactosylceramide
DIV: Days in vitro
