# Supplementary material for: Age-related increase in caveolin-1 expression facilitates cell-to-cell transmission of α-synuclein in neurons
Source: Mol Brain. 2021 Jul 28;14:122. doi: 10.1186/s13041-021-00834-2 (PMC8320051; doi:10.1186/s13041-021-00834-2)
Supplement: Supplementary file 1 — Additional file 1: Figure S1. Protein expression of stable cell line and the uptake of α-syn in cav-1 overexpressing SH-SY5Y cells. a EGFP only, WT cav-1-EGFP and Y14A cav-1-EGFP OE SH-SY5Y cells were lysed, and Western blot was performed with the indicated antibodies. b EGFP only, WT cav-1-EGFP and Y14A cav-1-EGFP OE SH-SY5Y cells were observed under confocal microscopy. c EGFP only and WT cav-1-EGFP OE SH-SY5Y cells were cocultured with differentiated α-syn OE SH-SY5Y cells cultured on the insert for 24 h. The cells were then lysed, and Western blot was performed with the indicated antibodies. The intensity of four independent experiments was analyzed. ** p < 0.01, unpaired t-test. Figure S2. Protein expression of stable cell line. a EGFP only, WT cav-1-EGFP or Y14A cav-1-EGFP OE SH-SY5Y cells were treated with 1 μM α-Syn fibrils for 10 min. The cells were then lysed, and Western blot was performed with the indicated antibodies. b A53T-E only, WT cav-1/A53T-E and Y14A cav-1/A53T-E OE SH-SY5Y cells were lysed, and Western blot was performed with the indicated antibodies. [file 13041_2021_834_MOESM1_ESM.pdf]

Additional file 1

Age-related increase in caveolin-1 expression facilitates cell-to-cell transmission of α-synuclein in neurons

Tae-Young Ha[1,2], Yu Ree Choi[1,2,3], Hye Rin Noh[1,2,3], Seon-Heui Cha[1,2,4], Jae-Bong Kim[1,2,3], and Sang Myun Park[1,2,3,*]

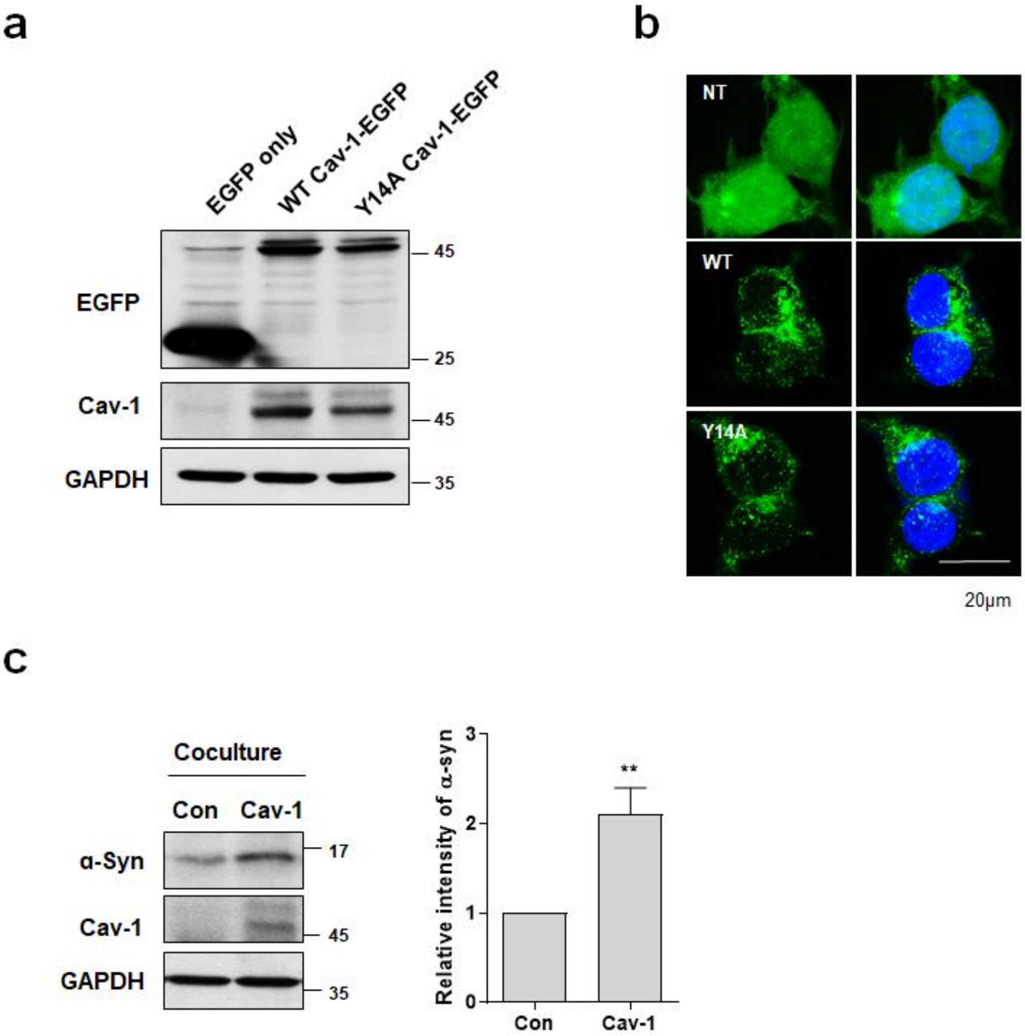

Figure S1. Protein expression of stable cell line and the uptake of α-syn in cav-1 overexpressing SH-SY5Y cells. a EGFP only, WT cav-1-EGFP and Y14A cav-1-EGFP OE SH-SY5Y cells were lysed, and Western blot was performed with the indicated antibodies. **b** EGFP only, WT cav-1-EGFP and Y14A cav-1-EGFP OE SH-SY5Y cells were observed under confocal microscopy. **c** EGFP only and WT cav-1-EGFP OE SH-SY5Y cells were cocultured with differentiated α-syn OE SH-SY5Y cells cultured on the insert for 24 h. The cells were then lysed, and Western blot was performed with the indicated antibodies. The intensity of four independent experiments was analyzed. ** $p < 0.01$, unpaired t-test.

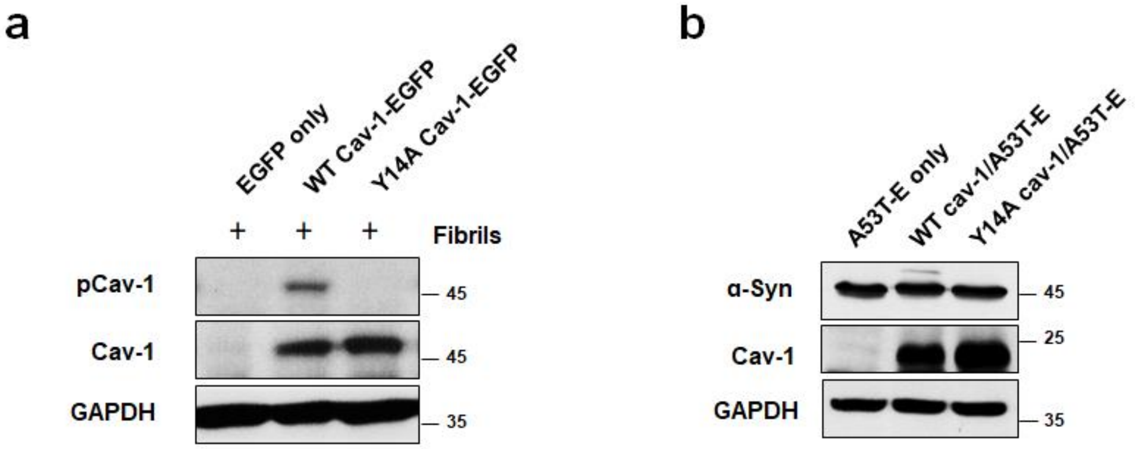

Figure S2. Protein expression of stable cell line. a EGFP only, WT cav-1-EGFP or Y14A cav-1-EGFP OE SH-SY5Y cells were treated with 1 μM α-Syn fibrils for 10 min. The cells were then lysed, and Western blot was performed with the indicated antibodies. **b** A53T-E only, WT cav-1/A53T-E and Y14A cav-1/A53T-E OE SH-SY5Y cells were lysed, and Western blot was performed with the indicated antibodies.
